# Vaccination card availability and childhood immunization in Senegal

**DOI:** 10.1186/s12889-020-08792-5

**Published:** 2020-05-12

**Authors:** Valérie Seror, Sébastien Cortaredona, Elhadji Yaya Ly, Samba Ndiaye, Ibrahima Gaye, Mouhamadou Fall, Patrick Peretti-Watel

**Affiliations:** 1 Aix Marseille Univ, IRD, AP-HM, SSA, VITROME, Marseille, France; 2 IHU-Méditerranée Infection, Marseille, France; 3 Agence nationale de la statistique et de la démographie, Rocade Fann Bel-air Cerf-volant, BP 116, Dakar, RP Sénégal; 4 Institut de Santé et de Développement, Université Cheikh Anta Diop (UCAD), B.P. 5005, Dakar - Fann, Dakar, Sénégal; 5 UNICEF – Sénégal, Route de l’Hôtel King Fahd Palace, BP 29720, Dakar, Sénégal; 6 ORS PACA, Southeastern Health Regional Observatory, 13006 Marseille, France

**Keywords:** Childhood vaccination, Vaccination cards, Coverage, Immunization, Socioeconomic, Senegal

## Abstract

**Background:**

The World Health Organization recommends recording vaccination status according to maternal recall in countries where administrative reporting systems are insufficiently reliable, as maternal recall in developing countries has been shown to be quite reliable compared with data from vaccination cards. This study aimed to investigate childhood vaccination coverage and its determinants according to the mothers’ presentation of vaccination cards.

**Methods:**

The data come from the 2017 Senegalese Demographic and Health Survey, a nationally representative household survey of women aged 15–49 years, with a questionnaire focusing on children’s health. This analysis was restricted to children aged 12–35 months (*n* = 4032) and it assessed vaccination coverage and associated sociodemographic factors with weighted multivariate logistic regressions. Stratified multivariate logistic regressions were also performed to investigate factors associated with routine childhood immunization uptake of the Bacillus Calmette-Guérin (BCG) vaccine, recommended for administration shortly after birth, as well as of the vaccines against yellow fever and measles (recommended at 9 months).

**Results:**

Comparison of vaccination coverage estimates according to the vaccination card or parental recall resulted in a 5–10% difference in estimated coverage for the BCG, pentavalent, measles, and yellow fever vaccines, but a huge difference for the polio vaccine (93.0% with the card, 32.0% without it). Presentation of the vaccination card was correlated with mothers’ attendance at health facilities (suggesting it serves as a concrete manifestation of a bond between mothers and the healthcare system) and their region of residence, but it was not correlated with usually strong predictors of childhood vaccination, such as maternal education level. Factors associated with vaccinations differed depending on whether they were administered shortly after birth or later on.

**Conclusions:**

Maternal recall was found to be quite reliable except for oral polio vaccination, which raises the possibility that complete immunization coverage rates could have been significantly underestimated due to potential confusion between injection and vaccination. Considering the ability to present vaccination cards as the materialization of a bond with the healthcare system, the decision path leading to vaccination among those who lack such a bond appears longer and more likely to be driven by supply-side effects.

## Background

Population-based surveys are a common and convenient tool for assessing vaccination coverage, especially in countries where administrative reporting systems are not sufficiently reliable; they are also useful in identifying factors associated with reported vaccination, related to either supply-side or demand-side effects [1, 2]. Evidence about vaccination in these surveys is usually derived from one of two main sources: home-based written vaccination records (typically vaccination card/booklet) or vaccination history, as recalled by the individual interviewed or, for a child, the child’s caretakers.

In developed countries, only a few studies rely exclusively on parental recall [3], and the lack of a written vaccination record is frequently considered an exclusion criterion for data analysis [4, 5] since parental recall of young children’s immunization histories has been found to be relatively poor [6–8]. Considerable discrepancies have been found between parental reports and healthcare providers’ records, and overreporting seems more frequent than underreporting [7]. Among the factors likely to affect parental recall, the specific vaccine considered and the child’s age (with declining recall accuracy as the child’s age increases) are most important [6, 8]. There are multiple possible sources of inaccurate reporting. Parents may simply forget a shot, which is likely to occur considering the high number of childhood vaccinations today and the similarity of the episodes in which they are administered within a limited period of time [8]. Conversely, parents may be reluctant to admit that their child was not vaccinated (social desirability bias). However, the main source of inaccurate reporting may relate to poor initial encoding of the memory of the relevant events during the interaction between parents and health professionals: parents may not understand the information provided about the vaccines being administered, or they may be too distracted to absorb this information properly. Conversely the health professionals involved in the vaccination process may not take the time to clearly identify the shots administered during the visit [7].

In developing countries, the picture is quite different, as home-based written vaccination records are frequently unavailable. In a recent study in Senegal, for example, of parents of a sample of children aged 12–23 months, parents of 31.6% of the children did not have a vaccination card for them. They were nevertheless included in the analysis, and their vaccination status was recorded according to maternal recall, as recommended by the World Health Organization [9]. Previous studies in sub-Saharan Africa and other low/middle income countries suggest that maternal recall is quite reliable compared with the data collected from vaccination cards [10, 11]. Nonetheless, the availability of these cards might be considered a relevant covariate in multivariate analyses conducted to identify factors associated with vaccination. The availability of immunization cards at the time of the survey, for example, turned out to be a strong predictor of complete immunization among children aged 12–23 months in studies in Senegal, Nigeria, and Ethiopia [12–14]. This finding is not surprising, as the possession of an immunization card (which is usually delivered during prenatal consultations) may be a proxy variable for unobservable factors strongly correlated with vaccinations, including the availability of health facilities or positive maternal attitudes toward vaccination [13].

This study presents a secondary analysis of the 2017 Demographic and Health Survey (DHS), a nationally representative household survey conducted by the Senegalese National Agency of *Statistics and* Demography (ANSD), which collected data about childhood vaccination coverage and its determinants [15]. We focused on immunization card availability to study its determinants and its impact on childhood vaccinations among children aged from 12 to 35 months. Specifically, we first compared vaccination coverage estimates based on either the vaccination card or parental recall only. Second, we investigated the sociodemographic factors associated with the presentation of a vaccination card. We expected that these factors would be similar to those usually associated with non-vaccination in African countries (hypothesis 1), including low level of maternal education, low household wealth, and rural residence [2, 9, 13, 14, 16–18]. Third, we investigated the sociodemographic factors associated with routine childhood immunization uptake of five vaccines, ranging from Bacillus Calmette-Guérin (BCG) vaccine, administered soon after birth, to yellow fever and measles vaccines, usually administered at 9 months. These analyses were stratified by the presentation of a vaccination card to the interviewer (yes/no), on the assumption that motivators and barriers to vaccination might differ between these two subsamples (hypothesis 2). We also expected that the patterns of determinants might change from the vaccines administered shortly after birth to those administered later (hypothesis 3).

## Methods

### Study setting & sampling design

Our data come from the DHS conducted in Senegal in 2017 [15] and downloaded from the DHS program website (dhsprogram.com). The DHS program was established by the United States Agency for International Development in 1984. Since then, more than 130 nationally representative DHS household-based surveys have been completed in about 70 countries. They are designed to collect data on various topics, including planning, reproductive health, and child health. Due to their subject matter, they focus on women of reproductive age (15–49), with two distinct questionnaires: a household questionnaire, the main purpose of which is to identify women with children under five and those eligible for individual interviews, and a woman’s questionnaire [19].

The study in Senegal in 2017 was a nationally representative household survey with a two-stage stratified cluster sampling design. In the first stage, the primary sampling units, which are the census districts, were selected with probability proportional to their population size. At the second stage, households were selected and enumerated within each area segment. The sample was stratified by urban and rural areas.

### Data collection & study population

Data collection started in April 2017 and ended that December. Overall, 8800 occupied households were selected and responded at a rate of 92% (*n* = 8522). Among the households interviewed, 17,586 women aged 15–49 years were eligible for the individual survey, and the response rate was 96% (*n* = 16,787).

All the women interviewed in the 2017 DHS sample in Senegal responded to a set of questions about the immunization coverage of their young children (aged under 36 months, *n* = 7011). As in previous studies devoted to childhood vaccination in various sub-Saharan African countries [9, 12–18], we restricted the analysis to children aged over 12 months at the time of the survey (*n* = 4709). Because a set of variables about the mothers’ healthcare use was only available for the youngest child of each woman (her last live birth), the final study population comprised 4032 children aged between 12 and 35 months at the time of the survey.

### Outcome variables & statistical analyses

Two data sources have been used to estimate vaccination coverage in the DHS studies: the vaccination card shown by mothers to interviewers and/or the mother’s recall of vaccination. For this study, we built a binary outcome, coded 0 or 1 (for the children for whom the mother showed the vaccination card to the interviewer). This study focused on the following vaccines: the Bacillus Calmette-Guérin (BCG) vaccine (recommended at birth), the pentavalent vaccine (for diphtheria, tetanus, pertussis, haemophilus influenza b, and hepatitis b, three doses recommended at 6, 10, and 14 weeks), the polio vaccine (an oral vaccine, three doses recommended at 6, 10, and 14 weeks), and the measles and yellow fever vaccines (both recommended at 9 months). Five binary outcomes were constructed for children’s immunization status, coded 0 or 1 (1 for those who received the single shot for the BCG, measles, and yellow fever vaccines; and 1 for those who completed all three shots for the pentavalent vaccine and all three oral doses for the polio vaccines). We also built a “complete immunization” outcome, coded 1 for children who received all five vaccines (0 otherwise).

To study the factors associated with the presentation of the vaccination card and immunization status, we retrieved the following information from the DHS database: the child’s characteristics (sex, age in months, and birth order), the mother’s characteristics (age at child’s birth, education level, and ethnic group) and her media exposure (watching TV, listening to radio, or reading newspapers), the household characteristics (wealth index), and geographical location (region of residence, rural/urban area).

The wealth index is a composite index of a household’s cumulative living standard that enables comparability between urban and rural areas. The downloaded DHS dataset included data related to the wealth index, calculated by principal component analysis (PCA) of the following items: source of drinking water, type of toilet, sharing of toilet facilities, material of principal floor, walls, roof, cooking fuel, household, services and possessions, such as electricity, TV, radio, watch, types of vehicles, quantity of agricultural land owned, and type and number of animals owned. The full methodology used to construct the wealth index is available [20] as are the principal component scores of each item included in the index; they can be found online (at https://www.dhsprogram.com/programming/wealth%20index/Senegal%20DHS%202017/Senegal%20DHS%202017.xlsx). As regards exposure to medias, we assumed that improved awareness and knowledge about vaccination due to greater media exposure [21–23] might result in a higher likelihood of vaccination card availability. Finally, variables on use of healthcare services, such as the place of delivery (home/health facility), antenatal care during pregnancy, and postnatal check-ups within 3 months were also retrieved from the DHS database.

Descriptive statistics including prevalence and frequency distributions were used to estimate children’s immunization coverage according to whether the vaccination card was presented to the interviewer or not. We used weighted univariate logistic regressions to identify factors that were associated with this presentation. Next, we ran multivariate analyses with a weighted logistic regression model. A stepwise approach was used to assess the iteration of variables and to control for potential confounders [24]. Finally, for each vaccination outcome (BCG, pentavalent, polio, measles, yellow fever, and complete immunization), two stepwise weighted logistic regression models were performed depending on whether or not the mother showed the vaccination card to the interviewer. Potential endogeneity with the dependent variables was checked with Housman’s test [25] (also known as either the Housman specification test or the Durbin, Hausman and Wu Test). Missing values were imputed to the category with the highest frequency in the multivariate models.

In the DHS survey, the sample is selected with unequal probability to expand the number of cases available for certain areas or subgroups for which statistics are needed. Thus, sampling weights were applied to all statistics to produce accurate representation as well as corrections for differential response rates for certain areas or subgroups. All analyses were based on two-sided *p*-values, with statistical significance defined by *p* ≤ 0.05. They were performed with SAS 9.4 statistical software (SAS Institute, Cary, NC, USA).

## Results

### Immunization coverage rates according to vaccination card availability

**Fig. 1:**
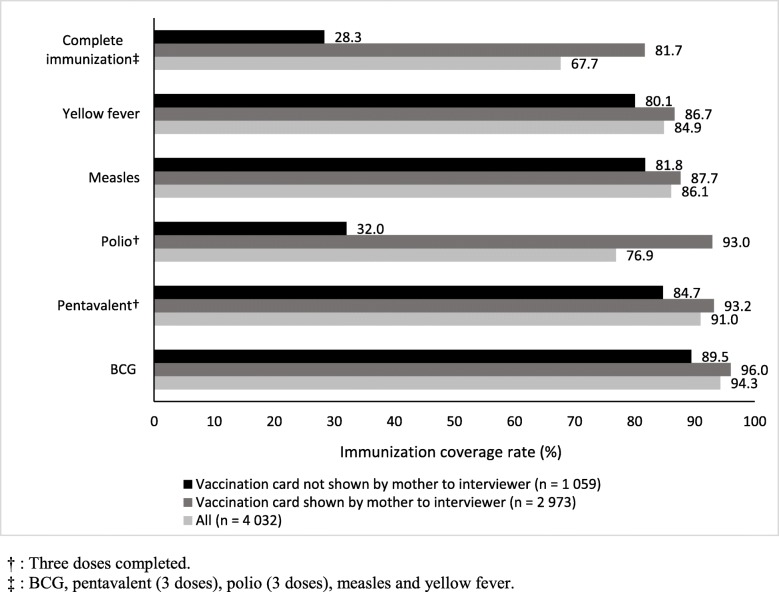
Immunization coverage of children aged 12–35 months according to the presentation of the vaccination card (Senegal DHS 2017 - *n* = 4032)

Figure 1 displays the estimated immunization coverage for each vaccine considered here, as well as for complete immunization, depending on whether the child’s mother showed the interviewer the vaccination card. Immunization coverage was systematically and significantly (*p* < 0.001) lower among the “no vaccination card” subsample. Moreover, although the difference in estimated coverage between these subsamples ranged between 5 and 10 percentage points for the BCG, pentavalent, measles, and yellow fever vaccines, it was huge for the polio vaccine (93.0% with the card, 32.0% without it). As a result, the difference was also huge for complete immunization (81.7% with vs 28.3% without the card).

### Factors associated with the presentation of the vaccination card

In the multivariate analyses, most of the factors related to either children’s or mothers’ characteristics had no statistically significant effect on the presentation of the vaccination card, except for the child’s age and mother’s media exposure: mothers of children aged 24–35 months were less likely to present a vaccination card (adjusted odds ratio (aOR) = 0.44) than mothers of children aged 12–23 months (see Table 1). Similarly, mothers who never watched TV, listened to the radio or read newspapers were less likely to have a vaccination card with them than those exposed to media. Two other significant factors were related to mothers’ attendance at health facilities: mothers who had attended antenatal care were more likely to present the vaccination card (aOR = 2.23), while those who gave birth at home (instead of at a health facility) were less likely to do so (aOR = 0.75). Finally, compared with women in northern Senegal, mothers interviewed in the western part of the country were more likely to have the vaccination card (aOR = 1.48) and those from the South less likely to do so (aOR = 0.73).

**Table 1:** Factors associated with the presentation of the vaccination card to the interviewer - weighted logistic regression (Senegal DHS 2017 - *n* = 4032)

	No vaccination card***n*** = 1059	Vaccination card***n*** = 2973	All ***n*** = 4032	OR 95% CI^**†**^	p	aOR 95% CI^**‡**^	*p*
	%	%	%				
**Age of the child at interview**							
12–23 months (ref.)	42.4	61.8	56.7	1		1	
24–35 months	57.6	38.2	43.3	0.45[0.39;0.52]	< 0.001	0.44[0.38;0.51]	< 0.001
**Sex of the child**							
Male (ref.)	51.2	51.1	51.1	1		NS	
Female	48.8	48.9	48.9	1.00[0.87;1.15]	0.961		
**Birth order**							
1 (ref.)	22.5	22.9	22.8	1		NS	
2	32.2	35.4	34.5	1.08[0.89;1.31]	0.443		
> 2	45.3	41.7	42.6	0.91[0.76;1.09]	0.281		
**Mother’s age at child birth**							
15–19	13.7	11.3	12.0	0.82[0.66;1.02]	0.080	NS	
20 (ref.)	48.5	49.0	48.8	1			
30+	37.8	39.7	39.2	1.04[0.89;1.21]	0.601		
**Mother’s educational level** (missing = 3)							
No education (ref.)	60.4	56.8	57.7	1		NS	
Primary	23.3	24.7	24.3	1.13[0.95;1.34]	0.161		
Secondary or higher	16.4	18.5	18.0	1.20[0.99;1.46]	0.060		
**Mother’s exposure to media**							
Not at all (ref.)	11.2	6.9	8.0	1		1	
Less than once a week	17.9	19.1	18.9	1.74[1.32;2.31]	< 0.001	1.51[1.13;2.02]	0.006
At least once a week	70.9	74.0	73.2	1.71[1.34;2.17]	< 0.001	1.23[0.94;1.60]	0.126
**Mother’s ethnic group**							
Wolof (ref.)	36.5	36.8	36.7	1		NS	
Puular	29.5	27.2	27.8	0.91[0.76;1.08]	0.295		
Serer	13.5	18.4	17.1	1.34[1.08;1.67]	0.008		
Manding	8.0	5.9	6.5	0.74[0.55;0.98]	0.035		
Other	12.5	11.7	11.9	0.93[0.74;1.17]	0.526		
**Household wealth quintile**							
Poorest	26.0	21.5	22.7	0.85[0.69;1.05]	0.135	NS	
Poorer	21.2	21.2	21.2	1.03[0.83;1.28]	0.774		
Middle (ref.)	20.9	20.3	20.5	1			
Richer	16.2	17.2	17.0	1.10[0.87;1.38]	0.439		
Richest	15.8	19.7	18.7	1.28[1.02;1.62]	0.033		
**Place of delivery**							
At health facility (ref.)	74.9	83.0	80.9	1			
At home	25.1	17.0	19.1	0.61[0.52;0.73]	< 0.001	0.75[0.62;0.91]	0.003
**Mother attended antenatal care** (missing = 84)							
No (ref.)	3.9	1.2	1.9	1		1	
Yes	96.1	98.8	98.1	3.24[2.04;5.13]	< 0.001	2.23[1.36;3.65]	0.001
**Postnatal check-up within 3 months** (missing = 6)							
No (ref.)	18.8	13.4	14.8	1		NS	
Yes	81.2	86.6	85.2	1.50[1.24;1.80]	< 0.001		
**Place of residence**							
Urban (ref.)	36.0	40.9	39.6	1		NS	
Rural	64.0	59.1	60.4	0.81[0.70;0.94]	0.005		
**Region**							
North (ref.)	17.1	16.3	16.5	1		1	
West	25.4	37.4	34.3	1.54[1.24;1.92]	< 0.001	1.48[1.18;1.86]	0.001
Center	31.1	28.7	29.3	0.96[0.78;1.19]	0.741	0.94[0.75;1.17]	0.562
South	26.3	17.6	19.9	0.70[0.56;0.88]	0.002	0.73[0.58;0.92]	0.008

†: Crude odds ratios with 95% confidence intervals (bivariate analysis)

‡: Adjusted odds ratios with 95% confidence intervals estimated using a weighted logistic regression model. A stepwise approach was used to assess the iteration of variables and to control potential confounders

*NS*: non statistically significant (*p* > 0.05) after stepwise selection

Note: Potential endogeneity with “antenatal care” was tested using the Housman’s test. The variables “distance to health facility” and “permission to go to health facility” were used as instrumental variables. No significant endogeneity issue was detected (*p* = 0.43)

### Presentation of the vaccination card and factors associated with childhood vaccinations

Household wealth was positively and statistically significantly associated with BCG vaccination, but only in the “vaccination card available” subsample (see Table 2). Conversely, maternal media exposure was positively correlated with BCG vaccination, but only in the “no vaccination card” subsample. Other significant factors included maternal attendance at health facilities, with the estimated effects much stronger among the “no vaccination card” subsample: delivery at home vs at a health facility (aOR = 0.60 in the ‘vaccination card available’ subsample, vs 0.32 in the ‘no vaccination card’ subsample), attendance at antenatal care (non-significant effect vs aOR = 5.42), and postnatal checkup (aOR = 1.81 vs aOR = 4.28). Similarly, the estimated effects for the region of residence were more significant in the “no vaccination card” subsample.

**Table 2:** Factors associated with vaccinations (BCG, pentavalent, and polio) of children aged 12–35 months - weighted logistic regressions (Senegal DHS 2017 - *n* = 4032)

	BCG				Pentavalent^**†**^				Polio^**†**^			
	Vaccination card ***n*** = 2973	p	No card ***n*** = 1059	p	Vaccination card ***n*** = 2973	p	No card ***n*** = 1059	p	Vaccination card ***n*** = 2973	p	No card ***n*** = 1059	p
	aOR 95% CI^†^		aOR 95% CI^‡^		aOR 95% CI^†^		aOR 95% CI^‡^		aOR 95% CI^†^		aOR 95% CI^‡^	
**Age of the child at interview** (ref. 12–23 mths												
24–36 months	NS		NS		NS		1.90[1.25;2.89]	0.003	NS		NS	< 0.001
**Sex of the child** (ref. male)												
Female	NS		NS		NS		NS		NS		NS	
**Birth order** (ref. 1)												
2	NS		NS		0.51[0.30;0.84]	0.008	NS		NS		NS	
3+					0.43[0.24;0.76]	0.004						
**Mother’s age at child birth** (ref. 20–30)												
15–19	NS		NS		0.43[0.26;0.72]	0.001	NS		NS		NS	
30+					1.38[0.96;1.99]	0.078						
**Mother’s education level** (ref. no education)												
Primary	NS		NS		1.07[0.73;1.56]	0.727	2.25[1.18;4.28]	0.013	0.85[0.60;1.21]	0.363	NS	
Secondary or higher					2.62[1.37;5.01]	0.004	2.31[1.02;5.23]	0.045	2.39[1.30;4.42]	0.005		
**Mother’s exposure to media**												
Less than once a week	NS		3.87[1.93;7.75]	< 0.001	1.23[0.74;2.05]	0.422	2.79[1.50;5.18]	0.001	NS		2.19[1.23;3.89]	0.007
At least once a week			4.26[2.35;7.72]	< 0.001	1.87[1.13;3.09]	0.015	3.39[1.98;5.81]	< 0.001			2.19[1.29;3.69]	0.003
**Mother’s ethnic group** (ref. Wolof)												
Puular	NS		NS		1.52[1.04;2.23]	0.031	NS		NS		0.47[0.32;0.69]	< 0.001
Serer					1.63[1.04;2.55]	0.032					1.06[0.70;1.60]	0.780
Manding					0.76[0.44;1.32]	0.337					0.58[0.32;1.06]	0.077
Other					1.32[0.78;2.23]	0.301					0.59[0.37;0.95]	0.029
**Household wealth quintile** (ref. middle)												
Poorest	0.42[0.23;0.77]	0.005	NS		1.06[0.68;1.65]	0.790	NS		1.08[0.71;1.65]	0.704	NS	
Poorer	0.64[0.34;1.20]	0.165			1.12[0.73;1.73]	0.606			1.20[0.80;1.81]	0.382		
Richer	1.47[0.60;3.57]	0.399			1.27[0.78;2.09]	0.338			1.32[0.82;2.15]	0.255		
Richest	3.57[1.00;12.72]	0.050			3.63[1.73;7.59]	0.001			4.12[1.92;8.81]	< 0.001		
**Place of delivery** (ref. at health facility)												
At home	0.60[0.39;0.92]	0.018	0.32[0.19;0.54]	< 0.001	NS		0.39[0.25;0.60]	< 0.001	0.67[0.47;0.95]	0.024	NS	
**Mother attended antenatal care** (ref. no)												
Yes	NS		5.42[2.14;13.72]	< 0.001	NS		3.74[1.53;9.14]	0.004	NS		NS	
**Postnatal check-up within 3 months** (ref. no)												
Yes	1.81[1.17;2.80]	0.008	4.28[2.57;7.14]	< 0.001	NS		4.62[2.94;7.24]	< 0.001	NS		NS	
**Place of residence** (ref. urban)												
Rural	NS		NS		NS		NS		NS		NS	
**Region** (ref. North)												
West	1.40[0.64;3.08]	0.397	24.65[1.43;426.24]	0.028	NS		2.58[0.87;7.71]	0.089	1.66[1.02;2.69]	0.040	0.43[0.28;0.67]	< 0.001
Center	0.69[0.39;1.21]	0.198	0.82[0.39;1.74]	0.600			0.44[0.23;0.84]	0.013	0.87[0.59;1.29]	0.502	0.53[0.35;0.81]	0.003
South	0.57[0.33;1.01]	0.052	0.31[0.15;0.61]	0.001			0.26[0.14;0.50]	< 0.001	1.03[0.66;1.61]	0.907	0.55[0.36;0.86]	0.008

Pentavalent vaccine uptake in the “vaccination card” sample was statistically significantly associated with numerous characteristics of the children, mothers, and households (respectively, for example, birth order; mother’s age at child’s birth, education level, media exposure, and ethnic group; wealth quintile), but not with any factor related to either attendance at health facilities or geographical location. Conversely, in the “no vaccination card” subsample, only three of any of the children’s, mothers’, or household characteristics considered had a significant effect on vaccination, namely, the child’s age at interview and the mother’s education level and media exposure. The main estimated effects were related to attendance at health facilities (place of delivery, attendance at antenatal care and at postnatal checkup), and geographical location.

The polio vaccine also showed distinct covariates in the two subsamples: maternal education, household wealth, and place of delivery in the “vaccination card” subsample, and in the “no card” subsample, her ethnic group, media exposure, and place of residence. We also found distinct effects for region of residence: living in western Senegal was positively associated with polio vaccination in the “vaccination card” subsample, compared with living in northern Senegal in the “no card” subsample.

Measles vaccine uptake, among other results, was higher among older children and those in the highest wealth index quintile in the “no card” subsample. Moreover, the positive effects of maternal education and media exposure were higher than in the “vaccination card” subsample (see Table 3). In both subsamples, we found that the odds of children being vaccinated were lower when mothers belonged to the Manding ethnic group. Moreover, only in the “no card” subsample did mothers’ failure to attend either antenatal care or the postnatal checkup and their place of delivery significantly increase the odds of the child’s vaccination. Finally, after we controlled for the other effects, children in the “vaccination card” subsample living in the Center region were less likely to be vaccinated.

**Table 3:** Factors associated with vaccinations (measles, yellow fever, complete immunization) of children aged 12–35 months - weighted logistic regressions (Senegal DHS 2017 - *n* = 4032)

	Measles				Yellow fever				Complete immunization^**†**^			
	Vaccinationcard ***n*** = 2973	p	No card ***n*** = 1059	p	Vaccination card ***n*** = 2973	p	No card ***n*** = 1059	p	Vaccination card ***n*** = 2973	p	No card ***n*** = 1059	p
	aOR 95% CI^†^		aOR 95% CI^‡^		aOR 95% CI^†^		aOR 95% CI^‡^		aOR 95% CI^†^		aOR 95% CI^‡^	
**Age of the child at interview** (ref. 12–23 mths												
24–36 months	NS		1.69[1.16;2.47]	0.007	1.44[1.14;1.82]	0.002	1.57[1.09;2.27]	0.015	1.36[1.11;1.67]	0.003	NS	
**Sex of the child** (ref. male)												
Female	NS		NS		NS		NS		NS		NS	
**Birth order** (ref. 1)												
2	NS		NS		NS		NS		NS		NS	
3+												
**Mother’s age at child birth** (ref. 20–30)												
15–19	0.62[0.44;0.88]	0.007	NS		NS		NS		NS		NS	
30+	0.79[0.62;1.00]	0.052										
**Mother’s education level** (ref. no education)												
Primary	1.37[1.02;1.84]	0.037	1.71[1.03;2.87]	0.040	1.46[1.09;1.94]	0.010	1.47[0.90;2.39]	0.122	1.12[0.87;1.44]	0.387	NS	
Secondary or higher	1.76[1.21;2.55]	0.003	4.24[1.85;9.71]	0.001	2.03[1.41;2.93]	< 0.001	3.89[1.77;8.58]	0.001	1.70[1.22;2.37]	0.002		
**Mother’s exposure to media**												
Less than once a week	1.45[0.96;2.20]	0.077	2.23[1.25;3.97]	0.007	1.33[0.88;2.00]	0.173	2.35[1.32;4.19]	0.004	1.25[0.87;1.80]	0.233	2.45[1.28;4.71]	0.007
At least once a week	1.90[1.30;2.78]	0.001	3.59[2.09;6.17]	< 0.001	1.71[1.17;2.48]	0.005	3.10[1.81;5.28]	< 0.001	1.55[1.09;2.21]	0.014	2.53[1.38;4.62]	0.003
**Mother’s ethnic group** (ref. Wolof)												
Puular	1.03[0.76;1.40]	0.831	0.42[0.25;0.71]	0.001	0.97[0.72;1.29]	0.818	0.57[0.35;0.94]	0.028	1.05[0.81;1.36]	0.709	0.42[0.28;0.63]	< 0.001
Serer	1.50[1.06;2.14]	0.023	0.74[0.37;1.44]	0.372	1.59[1.12;2.25]	0.009	0.89[0.47;1.69]	0.722	1.63[1.20;2.22]	0.002	1.16[0.76;1.77]	0.487
Manding	0.54[0.34;0.84]	0.007	0.25[0.12;0.50]	< 0.001	0.51[0.33;0.78]	0.002	0.36[0.18;0.70]	0.003	0.68[0.45;1.01]	0.057	0.63[0.34;1.17]	0.145
Other	0.91[0.61;1.37]	0.661	0.46[0.23;0.92]	0.027	0.93[0.62;1.37]	0.701	0.68[0.35;1.31]	0.248	1.20[0.83;1.72]	0.329	0.57[0.35;0.94]	0.027
**Household wealth quintile** (ref. middle)												
Poorest	NS		2.07[1.18;3.64]	0.011	NS		1.60[0.90;2.84]	0.110	0.86[0.64;1.17]	0.342	0.53[0.33;0.85]	0.014
Poorer			3.32[1.85;5.96]	< 0.001			2.44[1.37;4.33]	0.003	1.01[0.75;1.35]	0.960	0.98[0.64;1.50]	0.920
Richer			2.16[1.09;4.28]	0.027			2.99[1.49;5.98]	0.002	1.37[0.97;1.92]	0.071	1.33[0.83;2.10]	0.303
Richest			4.10[1.43;11.81]	0.009			4.89[1.82;13.17]	0.002	2.31[1.54;3.47]	< 0.001	1.29[0.79;2.11]	0.337
**Place of delivery** (ref. at health facility)												
At home	NS		0.45[0.29;0.69]	< 0.001	NS		0.34[0.23;0.52]	< 0.001	NS		NS	
**Mother attended antenatal care** (ref. no)												
Yes	NS		7.17[2.75;18.72]	< 0.001	NS		5.20[2.02;13.39]	< 0.001	NS		NS	
**Postnatal check-up within 3 months** (ref. no)												
Yes	NS		2.85[1.84;4.42]	< 0.001	1.39[1.04;1.85]	0.025	3.14[2.05;4.81]	< 0.001	1.35[1.04;1.75]	0.025	1.56[1.03;2.35]	0.337
**Place of residence** (ref. urban)												
Rural	NS		NS		NS		1.68[1.01;2.80]	0.047	NS		NS	
**Region** (ref. North)												
West	1.41[0.96;2.07]	0.078	NS		1.37[0.94;1.99]	0.098	NS		1.16[0.82;1.64]	0.404	0.40[0.26;0.63]	< 0.001
Center	0.64[0.45;0.92]	0.078			0.62[0.44;0.87]	0.006			0.74[0.55;1.00]	0.051	0.53[0.34;0.82]	0.004
South	1.07[0.72;1.59]	0.738			0.95[0.65;1.39]	0.806			0.90[0.65;1.26]	0.544	0.58[0.37;0.92]	0.022

†: Adjusted odds ratios with 95% confidence intervals (estimated using a weighted logistic regression model) among mothers who presented the vaccination card to the interviewer

‡: Adjusted odds ratios with 95% confidence intervals (estimated using a weighted logistic regression model) among mothers who did not present the vaccination card to the interviewer

*NS* non statistically significant (*p* > 0.05) after stepwise selection

Yellow fever vaccination was more frequent in older children in both subsamples. Results for maternal media exposure and ethnic group and for household wealth and region of residence were the same as for measles vaccination. Moreover, the positive impact of maternal attendance at health facilities (place of delivery, antenatal care, and postnatal checkup) was much stronger in the “no card” sample.

Finally, the significant effects for complete immunization were very similar to those estimated for the polio vaccine in both subsamples, including the regional effects.

## Discussion

### Main results

Vaccination coverage estimates were significantly higher among children whose mothers showed the vaccination card to the interviewer than for those whose mothers did not. This difference was dramatic for polio vaccination (93.0% vs 32.0%) and necessarily produced a similar gap for the estimates of complete immunization coverage. In the multivariate analyses, most of the factors related to characteristics of children, mothers, and households had no influence on presentation of the vaccination card to the interviewer, with the notable exception of maternal media exposure. The main positive effects were related to attendance at healthcare facilities and region of residence (western vs. southern Senegal). The factors significantly associated with successive vaccinations differed depending on the subsample considered (with or without the vaccination card). These factors also varied across vaccinations, except for similarities between measles and yellow fever vaccinations on the one hand, and between polio and complete immunization on the other.

### Study limitations

Apart from the usual limitations of self-reported surveys, the main limitation of this study is that some time-varying sociodemographic factors were measured at the time of the survey, i.e., not at the time of each vaccination considered. As a result, statistical relations between sociodemographic characteristics and vaccinations should be interpreted cautiously. For example, a household in the fifth quintile of the wealth index at the time of the survey might have been in a lower quintile when the child received the BCG vaccine. Similarly, inability to show the child’s vaccination card to the interviewer at the time of the survey does not necessarily mean that the card was permanently lost or that it had been unavailable in the previous months when vaccinations had been scheduled. Finally, we should mention a potential bias in vaccination coverage estimates, due to child mortality, to which vaccine-preventable diseases contribute, and the DHS does not collect data on deceased children.

### Estimated immunization coverage rates and presentation of the vaccination card

The fact that estimated immunization coverage rates were lower in the “no card” subsample is consistent with previous studies that have found mothers’ ability to present this card to be a strong predictor of complete childhood immunization [12–14]. Nonetheless, contrary to hypothesis 1, not having it readily available was not correlated with other usually strong predictors of childhood vaccination, notably maternal education level, but it was correlated with mothers’ attendance at health facilities and their region of residence. In particular, presentation of the vaccination card was more frequent in western Senegal, where the estimated immunization coverage rates are highest [16].

Nevertheless, the enormous gap observed for polio vaccination - 93% among those with vaccination cards and only 32% among the “no card” subsample (and markedly different from the 5 to 10 percentage-point gap for the other vaccines) - is quite disturbing, and a strong reporting bias related to this vaccination may be suspected. As we pointed out in the introduction, a main source of inaccurate reporting of past vaccinations is likely to be poor initial encoding of the memory of the relevant events [7]. In this case, and contrary to most other childhood vaccines used in Senegal, which are injected, the polio vaccine is taken orally as drops. Although this route of administration is common to many treatments in both allopathic and traditional medicine, previous studies have shown that laypeople frequently confuse injection with vaccination since they tend to consider that vaccination always involves an injection [26–28]. This might well explain at least in part the considerable discrepancies observed between maternal recall and data from vaccination cards. As a result, parents’ misunderstanding of polio vaccine leads not only to underestimated vaccination coverage among children whose mothers were not able to present their vaccination card to interviewers but also to a massive mechanical underestimation of complete immunization coverage.

### Presentation of the vaccination card and factors associated with vaccinations

As expected, the factors associated with childhood vaccinations varied according to whether mothers showed the vaccination card to interviewers (hypothesis 2 confirmed). Ability to present the child’s vaccination card at the time of the survey correlated with mothers’ attendance at health facilities, perhaps suggesting that it is a concrete manifestation of a bond between mothers and the health system. While the decision path leading to vaccination is likely to differ according to whether such a bond exists, our results suggest that this pathway might be longer when it does not. In the “no card” subsample, older children (aged 24–35 months) were indeed more likely to receive the pentavalent, measles, and yellow fever vaccines (vs. only the last one in the other subsample), indicating delayed vaccinations that are suboptimal from a public health point of view [2]. Thus, interventions designed to improve the retention of child immunization cards may help improving timely childhood vaccination coverage [29].

In addition, the factors associated with vaccinations differed depending on whether vaccines were administered soon after birth or later: hypothesis 3 was confirmed. This finding, obtained in both subsamples, is consistent with one crucial aspect of contemporary vaccine hesitancy, that people are supposed to endorse vaccine-specific attitudes and behaviors [30, 31]. Despite the differences reported, some common patterns emerged from our results. Among the children whose mothers had had the vaccination card available for presentation, characteristics of the child, mother, and household were stronger determinants of vaccination, whereas geographical location and mothers’ attendance at healthcare facilities were stronger determinants of vaccination in the “no card” subsample. One notable exception was maternal media exposure, which was a stronger predictor of vaccination in the “no card” subsample. Nevertheless, watching TV, listening to the radio, and/or reading are also indicators of exposure to prevention campaigns that promote vaccination on these media [21, 22] and may thus be considered a supply-side effect.

In other words, when there was a bond between the family and the health system, embodied by the ability to present the vaccination card, vaccinations were mainly driven by demand-side effects that illustrated the social differentiation of vaccination-related behaviors. Conversely, in the absence of such a bond, vaccinations tended to be mainly driven by supply-side effects. According to the theoretical framework proposed by the WHO to understand contemporary vaccine hesitancy [30, 31], this hesitancy is a matter of complacency, convenience, and confidence. In our study, however, convenience issues probably weighed most heavily on inability to present the vaccination card.

## Conclusions

In developing countries, quantitative surveys conducted to estimate childhood vaccination coverage rely on maternal recall when mothers are unable to present their child’s vaccination card to interviewers. Using secondary analysis of the 2017 DHS conducted in Senegal, this study aimed to investigate childhood vaccination coverage and its determinants according to the mothers’ presentation of vaccination cards, as well as the determinants of such presentation. We would like to highlight the following points. First, polio vaccine coverage as well as complete immunization coverage are likely to be greatly underestimated in Senegal as well as in other countries were the polio vaccine is taken orally. Secondly, the decision path leading to vaccination appeared longer when mothers were unable to present their child’s immunization card, thus interventions designed to improve the retention of these cards may help improving timely childhood vaccination coverage. Thirdly, the patterns of vaccination determinants depended on which vaccine was considered, which is consistent with contemporary vaccine hesitancy.

## Data Availability

The 2016 DHS data are available to the general public by request from the Measure DHS website [http:// www. dhsprogram.com]. We submitted a request to the DHS program by describing the purpose and objectives of the study and thereafter received permission to download the dataset.

## Abbreviations

DHS: Demographic and Health Survey
ANSD: Senegalese National Agency of *Statistics and* Demography
BCG: Bacillus Calmette-Guérin
PCA: Principal Component Analysis
aOR: Adjusted Odds Ratio
WHO: World Health Organization

## References

[1] World Health Organization. World Health Organization vaccination coverage cluster surveys: reference manual. No. WHO/IVB/18.09. World Health Organization; 2018.

[2] Mbengue MAS, Mboup A, Ly ID, Faye A, Camara FBN, Thiam M, Ndiaye BP, Dieye TN, Mboup S (2017). Vaccination coverage and immunization timeliness among children aged 12–23 months in Senegal: a Kaplan-Meier and Cox regression analysis approach. Pan Afr Med J.

[3] Bocquier A, Ward J, Raude J, Peretti-Watel P, Verger P (2017). Socioeconomic differences in childhood vaccination in developed countries: a systematic review of quantitative studies. Expert Rev Vaccines.

[4] Steyer TE, Mainous AG, Geesey ME (2005). The effect of race and residence on the receipt of childhood immunizations: 1993–2001. Vaccine.

[5] Poethko-Müller C, Ellert U, Kuhnert R, Neuhauser H, Schlaud M, Schenk L (2009). Vaccination coverage against measles in German-born and foreign-born children and identification of unvaccinated subgroups in Germany. Vaccine..

[6] Suarez L, Simpson DM, Smith DR (1997). Errors and correlates in parental recall of child immunizations: effects on vaccination coverage estimates. Pediatrics.

[7] Willis G, Tourangeau R. Response errors in surveys of children's immunizations. Vol. 6. US Department of Health & Human; 1999.

[8] Ezzati-Rice TM, Zell ER, Massey JT, Nixon MG (1996). Improving the assessment of vaccination coverage rates with the use of both household and medical provider data. Proceedings of the Section on Survey Research Methods.

[9] Mbengue MAS, Sarr M, Faye A, Badiane O, Camara FBN, Mboup S (2017). Determinants of complete immunization among senegalese children aged 12-23 months: evidence from the demographic and health survey. BMC Public Health.

[10] George K, Victor SAR (1990). Reliability of mother as an informant with regard to immunisation. Indian J Pediatr.

[11] Gareaballah ETLB (1989). The accuracy of mother’s report about their children vaccination status: WHO bulletin. Bull World Health Organ.

[12] Ndiaye NM, Ndiaye P, Diédhiou A, Guèye AS, Tal-Dia A (2009). Factors related to failure to complete immunization of children aged 10-23 months in Ndoulo (Senegal). Sante Montrouge Fr.

[13] Fatiregun AA, Okoro AO (2012). Maternal determinants of complete child immunization among children aged 12-23 months in a southern district of Nigeria. Vaccine.

[14] Lakew Y, Bekele A, Biadgilign S (2015). Factors influencing full immunization coverage among 12-23 months of age children in Ethiopia: evidence from the national demographic and health survey in 2011. BMC Public Health.

[15] Agence Nationale de la Statistique et de la Démographie (2018). Sénégal : Enquête Démographique et de Santé Continue (EDS-Continue) 2017.

[16] Agence Nationale de la Statistique et de la Démographie (2017). Sénégal : Enquête Démographique et de Santé Continue (EDS-Continue) 2016.

[17] Russo G, Miglietta A, Pezzotti P, Biguioh RM, Bouting Mayaka G, Sobze MS, Stefanelli P, Vullo V, Rezza G (2015). Vaccine coverage and determinants of incomplete vaccination in children aged 12–23 months in Dschang, West Region, Cameroon: a cross-sectional survey during a polio outbreak. BMC Public Health.

[18] Seck I, Faye A, Mbacké Leye MM, Bathily A, Camara MD, Ndiaye P (2012). Measles epidemic and response in the region of Dakar (Senegal) in 2009. Sante Publique Vandoeuvre-Nancy Fr.

[19] Rutstein SO, Rojas G (2006). Guide to DHS statistics.

[20] Rutstein SO (2017). DHS Wealth Index Construction.

[21] Ortiz RR, Smith A, Coyne-Beasley T (2019). A systematic literature review to examine the potential for social media to impact HPV vaccine uptake and awareness, knowledge, and attitudes about HPV and HPV vaccination. Hum Vaccines Immunother.

[22] Hilton S, Hunt K, Langan M, Bedford H, Petticrew M (2010). Newsprint media representations of the introduction of the HPV vaccination programme for cervical cancer prevention in the UK (2005–2008). Soc Sci Med 1982.

[23] Kelly BJ, Leader AE, Mittermaier DJ, Hornik RC, Cappella JN (2009). The HPV vaccine and the media: how has the topic been covered and what are the effects on knowledge about the virus and cervical cancer?. Patient Educ Couns.

[24] Hosmer D, Lemeshow S (1980). A goodness-of-fit test for the multiple logistic regression model. Commun Statistics.

[25] Wooldridge JM (2011). Econometric Analysis of Cross Section and Panel Data – Solutions Manual and Supplementary Materials 2e.

[26] Jaffré Y, Bonnet D, Jaffré Y (2003). Transmissions, prudences et préventions en pays mande. Les maladies de passage.

[27] Alfieri C, Bonnet D, Jaffré Y (2003). Connaissances populaires et pratiques de prevention des infections respiratoires aiguës infantiles en population bobo (Burkina Faso). Les maladies de passage.

[28] Dagobi AE, Bonnet D, Jaffré Y (2003). La gestion locale des épidémies dans la vallée du fleuve Niger. Les maladies de passage.

[29] Asres M, Tessema F. Contribution of plastic bags to the retention of child immunization cards in Gambella Region and Assosa Zone, Benishangul-Gumuz Region. Ethiop J Health Dev. 2019;33.

[30] Dubé E, Laberge C, Guay M, Bramadat P, Roy R, Bettinger J (2013). Vaccine hesitancy: an overview. Hum Vaccines Immunother.

[31] Noni E (2015). MacDonald, the SAGE working group on vaccine hesitancy. Vaccine hesitancy: definition, scope and determinants. Vaccine..

